# Fungi Identify the Geographic Origin of Dust Samples

**DOI:** 10.1371/journal.pone.0122605

**Published:** 2015-04-13

**Authors:** Neal S. Grantham, Brian J. Reich, Krishna Pacifici, Eric B. Laber, Holly L. Menninger, Jessica B. Henley, Albert Barberán, Jonathan W. Leff, Noah Fierer, Robert R. Dunn

**Affiliations:** 1 Department of Statistics, North Carolina State University, Raleigh, North Carolina, United States of America; 2 Department of Applied Ecology, North Carolina State University, Raleigh, North Carolina, United States of America; 3 Department of Biological Sciences, North Carolina State University, Raleigh, North Carolina, United States of America; 4 Cooperative Institute for Research in Environmental Sciences, University of Colorado, Boulder, Colorado, United States of America; 5 Department of Ecology and Evolutionary Biology, University of Colorado, Boulder, Colorado, United States of America; Vanderbilt University, UNITED STATES

## Abstract

There is a long history of archaeologists and forensic scientists using pollen found in a dust sample to identify its geographic origin or history. Such palynological approaches have important limitations as they require time-consuming identification of pollen grains, a priori knowledge of plant species distributions, and a sufficient diversity of pollen types to permit spatial or temporal identification. We demonstrate an alternative approach based on DNA sequencing analyses of the fungal diversity found in dust samples. Using nearly 1,000 dust samples collected from across the continental U.S., our analyses identify up to 40,000 fungal taxa from these samples, many of which exhibit a high degree of geographic endemism. We develop a statistical learning algorithm via discriminant analysis that exploits this geographic endemicity in the fungal diversity to correctly identify samples to within a few hundred kilometers of their geographic origin with high probability. In addition, our statistical approach provides a measure of certainty for each prediction, in contrast with current palynology methods that are almost always based on expert opinion and devoid of statistical inference. Fungal taxa found in dust samples can therefore be used to identify the origin of that dust and, more importantly, we can quantify our degree of certainty that a sample originated in a particular place. This work opens up a new approach to forensic biology that could be used by scientists to identify the origin of dust or soil samples found on objects, clothing, or archaeological artifacts.

## Introduction

In the Sherlock Holmes mysteries the author Arthur Conan Doyle repeatedly has Holmes solve crimes by identifying the geographic origin of mud on a shoe, a pair of pants or some other material [1]. Since the publication of the Sherlock Holmes mysteries, there has been a large body of research devoted to determining when and where a dust sample might have originated [2–4]. Such work has been valuable in both archaeological and criminal investigations and has been used to ascertain the origin of dust or soil found on artifacts [1], on skin, in lungs [2], on clothes, on a document [5] or contraband in a shipment [6] or even on the grill of a car.

Such investigations can be based on the abiotic characteristics of the soil or dust [7], as Sherlock described, but they are frequently based on analyses of the types of plant pollen present in such samples. Because the plant communities found in different regions and habitats are often distinct, the idea is that plant pollen found in dust samples can be used to identify where that dust sample originated. Yet, while the idea of “biogeoprinting” samples on the basis of pollen samples is beguiling it has suffered from several problems that have hindered its utility for forensic dust analysis [8–10]. First, it requires that samples be sufficiently rich in pollen to permit identification. Second, it requires that the collected pollen be diagnostic not just of a particular biome (e.g., temperate forest) but of a more specific region, the odds of which improve with the number of pollen taxa encountered [11]. Third, it requires that we can accurately identify the pollen from all over the world or at least all over the region from which an object or body has potentially originated, which requires comprehensive collections of pollen [12] and the expertise necessary to match pollen to those collections [13]. In practice, this has meant that, while the use of palynological approaches in forensics and archaeology has become more common [4], it has depended heavily on expert identification of the pollen of individual plant species and knowledge about the distribution of those species. As a result, palynology remains, as a recent review put it, “a rarely used technique,” [14] relative to many of the other tools available to crime scene investigators or archaeologists. Moreover, when palynology is used to discern the geographic origin of a sample, the result is almost always based on expert opinion and devoid of statistical inference.

In addition to pollen, dust typically harbors a myriad of other taxa, including large numbers of microbial taxa [15] and there is some evidence that the analysis of these taxa can also be used to identify the geographic origin of dust or soil samples [16, 17]. Fungi represent a particularly promising taxon for biogeoprinting and forensic analyses in general [18] for a variety of reasons. First, the global diversity of fungi is enormous with nearly 100,000 described fungal species and far more that remain undescribed [19] with individual samples of soil or dust harboring large numbers of fungal taxa [20, 21]. Second, many fungal taxa are restricted in their geographic distribution and are only found in particular locations or ecosystem types [22]. Third, since many fungal taxa produce spores that are tolerant of dessication and other environmental stresses they can persist in samples for prolonged periods of time. Despite the potential advantages of fungal analyses for biogeoprinting, their utility remains more potential than realized and there are very few cases of fungi being used for forensic investigations [18]. We do not know if fungi can effectively be used to determine the geographic origin of dust samples and the utility of such an approach will ultimately require combining a broad-scale analysis of dust-associated fungi with rigorous statistical analyses to assess the probability that a sample has come from a particular habitat or region.

Working with the public, we obtained outdoor dust samples from 928 sites located across the United States. We identified the fungal taxa present in each of the collected samples via amplicon sequencing on the Illumina HiSeq platform of an internal transcribed spacer (ITS) region of the rRNA operon. We then took a random subset of these samples and developed a statistical learning model via discriminant analysis [23] based on fungal taxa occurrences across the country. Using the remaining samples (a set independent from the first group), we tested whether the model could predict the geographic area from which a given dust sample was collected. An error was assigned to each sample on the basis of the great-circle distance in kilometers between its actual location and its predicted location. This prediction procedure was repeated across disjoint subsets of the 928 samples via five-fold cross-validation. The resulting model successfully predicts the origin of samples to within a great-circle distance of 230 km, on average, with high probability.

## Methods

### Data collection

Outdoor dust samples were collected by volunteers participating in the Wild Life of Our Homes project [24] (WLOH, homes.yourwildlife.org), a continental-scale citizen science project mapping microbial diversity (fungi, bacteria, archaea) inside and outside the home. We recruited participants, representing all 50 states and the District of Columbia, through our website, social media and email campaigns over the period January 2012 to March 2013. The 1,430 enrolled participants were provided a written Informed Consent form approved by the North Carolina State University’s Human Research Committee (Approval No. 2177) as well as instructions for sampling their home and a home microbe sampling kit. Each home sampling kit contained dual-tipped sterile BBL CultureSwabs. Participants were instructed to sample the upper door trim on the outside surface of an exterior door, a sampling location that is unlikely to be cleaned frequently and serves as a passive collector of outdoor aerosols and dust with little to no direct contact from the home occupants. Here we focus on *n* = 928 of the 1430 samples that were successfully sequenced in our lab.

### Molecular analysis

Participants returned swabs by first-class mail over the period March 2012 to May 2013, and these swabs were stored in a -20°C freezer until processed. Latitude and longitude coordinates were derived from location information (address) and were used to obtain geo-referenced environmental variables for each household. Daily temperature (°C) and daily precipitation (mm) were calculated from the Climate Research Unit Time Series v3.21 Dataset [25] (monthly coverage from 1901 to 2009) and land type was classified by the National Land Cover Database 2006 [26] as belonging to one of twenty classes respresenting varying types of urbanization, forestation, wetlands, etc. As fungal communities likely differ between varied climates [22], accounting for these data may identify climates for which our forthcoming model excels or struggles at making accurate spatial origin predictions. This analysis is useful because it would allow one to make predictions about the types of fungi likely to be found in samples from a particular region without necessarily collecting lots of samples from that region.

Swabs were prepared for sequencing using the direct PCR approach [27]. Swab tips were placed directly into wells in 2 mL 96-well plates (Axygen Inc.) along with the appropriate negative control samples. Plates were processed using the Extract-N-Amp PCR kit (Sigma-Aldrich, Inc.) following a modified version of the manufacturers instructions. After each well received 250 *μ*L of the Extract-N-Amp Extraction solution, the plate was sealed securely with a 96 round well Impermamat Silicon Sealing Mat (Axygen, Inc.) and heated at 90°C for 10 minutes in a dry bath. Extract-N-Amp Dilution solution was then added to the wells at a 1:1 ratio to the Extraction solution and mixed gently by pipetting. The plate was resealed with the mat and stored at 4°C. PCR was conducted in 20 *μ*L triplicate reactions per sample using 10 *μ*L of Extract-N-Amp Ready Mix, 1 *μ*L of the forward and reverse primers, 5 *μ*L of PCR-grade water, and 4 *μ*L of the Extract-N-Amp sample solutions from the 96-well plate.

Fungal diversity in each sample was assessed using a high-throughput sequencing method to characterize the variation in a marker gene sequence. We sequenced the first internal transcribed spacer (ITS1) region of the rRNA operon, the most widely used ‘barcode’ for fungal community analyses [28], using the ITS1-F (CTTGGTCATTTAGAGGAAGTAA) and ITS2 (GCTGCGTTCTTCATCGATGC) primer pair [21]. The primers included the appropriate Illumina adapters with the reverse primers also having an error-correcting 12-bp barcode unique to each sample to permit multiplexing of samples. PCR products from all samples were quantified using the PicoGreen dsDNA assay, and pooled together in equimolar concentrations. Samples were sequenced on an Illumina HiSeq instrument. All sequencing runs were completed at the University of Colorado Next Generation Sequencing Facility.

The 100-bp sequences were demultiplexed using a custom Python script with quality filtering and phylotype (i.e., operational taxonomic unit) clustering conducted using the UPARSE pipeline [29]. During quality filtering, a maxee value of 0.5 was used (indicating that on average 0.5 nucleotides were incorrectly assigned in every sequence). Sequences were also dereplicated and singleton sequences were removed prior to phylotype determination. Representative sequences from the phylotypes were checked for ≥ 75% similar to ITS1 sequences contained in the UNITE November, 2012 database [30], the most comprehensive reference database for sequence-based fungal analyses. We discarded sequences that were < 75% similar to those in the UNITE database because, while they could be fungal, they could also be from other non-fungal, eukaryotic groups and we wanted to be careful to restrict our analyses just to those taxa that we were very confident were fungal. The representative sequences were then used to categorize the raw sequences into phylotypes at the 97% similarity threshold. Phylotypes were classified to taxonomic groups using the RDP classifier with a confidence threshold of 0.5 [31] against the UNITE database. Samples were rarefied to 20,000 randomly-selected sequences per sample in order to compare all samples at an equivalent sequencing depth.

### Statistical analysis

Spatial analysis and classification of the sequenced fungal taxa data was achieved via discriminant analysis [23]. Discriminant analysis is a two-stage classification method built on Bayes’ Theorem that classifies a new observation as arising from one of many possible populations based on its measured characteristics. The analysis proceeded in two stages: we first estimated the spatial distribution of each species’ occurrence probability using available samples, and then inverted these probabilities to predict the spatial origin of a new sample. Define *Y*_*ij*_ as the binary indicator that species *j* = 1,…,*m* is present in the sample taken at spatial location **s**_*i*_, *i* = 1,…,*n*, and let *p*_*j*_(**s**_*i*_) = Prob(*Y*_*ij*_ = 1).

We estimated the probability of the presence of species *j* in samples taken across a fine grid of points 𝓣 covering the continental United States using kernel smoothing [23]. Kernel smoothing locally weights noisy observations via a Gaussian kernel and thereby produces a smooth portrait of estimated occurrence probabilities over 𝓣, allowing for our method to make predictions at locations for which we may not have dust sample data. We let 𝓣 = {**t**_1_, …, **t**_*N*_} with larger choices of *N* achieving finer granularity at the expense of increased computational costs. The estimated occurrence probability of species *j* at location **t** ∈ 𝓣 is

$$\begin{array}{c} {\hat{p}}_{j}\left(\text{t}\right)=\sum_{i=1}^{n}w_{ij}\left(\text{t}\right)Y_{ij}\;\text{where}\;w_{ij}\left(\text{t}\right)=\frac{k_{j}(||\text{t}-{\text{s}}_{i}\left||\right)}{\sum_{l=1}^{n}k_{j}(||\text{t}-{\text{s}}_{l}\left||\right)}, \end{array}$$ (1)

∣∣**t** − **s**_*i*_∣∣ is the great-circle distance (km) between **t** and **s**_*i*_, $k_{j}(h)=\exp[-h^{2}{\left(2\rho_{j}^{2}\right)}^{-1}]$ is the Gaussian kernel function, and *ρ*_*j*_ is the kernel bandwidth. The estimated probability ${\hat{p}}_{j}(\text{t})$ is a locally-weighted average of the observations, with the weights *w*_*ij*_(**t**) decaying as a function of the distance from **t**.

We select kernel bandwidths separately for each species via generalized cross-validation [23]. For species *j* = 1,…,*m*, define **y**_*j*_ = (*Y*_1*j*_,…,*Y*_*nj*_)^*T*^ and **W**_*ρ*_ = [**W**(**s**_1_),…,**W**(**s**_*n*_)]^*T*^ with **W**(**s**_*i*_) = [*w*_1*j*_(**s**_*i*_),…,*w*_*nj*_(**s**_*i*_)]^*T*^, *i* = 1,…,*n*, so that ${\hat{\text{y}}}_{j}={\text{W}}_{\rho }{\text{y}}_{j}$. Then the best kernel bandwidth *ρ*_*j*_ for species *j* minimizes

$$\begin{array}{c} GCV\left(\rho \right)=\frac{\frac{1}{n}{\left({\hat{\text{y}}}_{j}-{\text{y}}_{j}\right)}^{T}\left({\hat{\text{y}}}_{j}-{\text{y}}_{j}\right)}{{\left[1-trace\left({\text{W}}_{\rho }\right)/n\right]}^{2}}\cdot \end{array}$$ (2)

Using *ρ*_*j*_ and (Eq 1) we find ${\hat{\text{p}}}_{j}={\left[{\hat{p}}_{j}({\text{t}}_{1}),\ldots ,{\hat{p}}_{j}({\text{t}}_{N})\right]}^{T}$, the estimated spatial distribution of occurrence probabilities of species *j* over 𝓣.

Given these occurrence probabilities for every species, we then classified the spatial origin of a new sample with binary features (*Y*_01_,…,*Y*_0*m*_) taken at an unknown location **s**_0_. Assume a flat prior is placed on 𝓣 so that a sample is equally likely to have originated from any location **t**. Then the Bayes’ rule (under 0/1 loss) is

$$\begin{array}{ccc} \hat{s} & = & \underset{\text{t}\;\in \;T}{\arg\;\max}\;\log L\left(\text{t}|Y_{01},\ldots ,Y_{0m}\right),\;\text{where} \end{array}$$ (3)

$$\begin{array}{ccc} \log L(\text{t}|Y_{01},\ldots ,Y_{0m}) & = & \sum_{j=1}^{m}Y_{0j}\log\left[{\hat{p}}_{j}\left(\text{t}\right)\right]+\left(1-Y_{0j}\right)\log\left[1-{\hat{p}}_{j}\left(\text{t}\right)\right]\cdot \end{array}$$ (4)

That is, the Bayes’ rule selects the spatial location that maximizes the log-likelihood of the new sample.

We performed five-fold cross-validation to illustrate the method. The data were randomly split into five groups. For each group, its data comprised the testing data, and the data in the remaining four groups formed the training data. The training data were split further into subtraining (80% of the training data) and subtesting (20% of the training data). With the subtraining data, we obtained estimated occurrence probabilities via (Eq 1) with per-species bandwidths minimizing (Eq 2). Then, for each sample in the testing data, we inverted these probabilities with (Eq 3) to predict their spatial origins. The great-circle distance in kilometers between a sample’s predicted origin $\hat{\text{s}}$ and its true origin **s**_0_ defines prediction error.

Suppose, however, that rather than predict a single spatial location $\hat{\text{s}}$ as the true origin **s**_0_, it is of interest to form a neighborhood around $\hat{\text{s}}$ that contains **s**_0_ with some degree of certainty. Consider a sample collected from an unknown location **s**_0_ with features (*Y*_01_,…,*Y*_0*m*_) from the withheld subtesting data, and assume a flat prior on 𝓣. Using the per-species bandwidths found with the subtraining data, we calculate (Eq 4) at every **t** ∈ 𝓣. Standardizing these log-likelihood values so that they sum to one yields a predictive probability mass function, say, *f* over 𝓣 such that *f*(**t**) expresses the probability the sample originates from **t**. We threshold these probabilities to form a neighborhood

$$\begin{array}{c} R_{q}=\left\{\text{t}\in T:f\left(\text{t}\right)>c\right\}\;\text{where}\;c\;\text{is}\;\text{the}\;\text{value}\;\text{so}\;\text{that}\;\sum_{\text{t}\in R_{q}}f\left(\text{t}\right)=q\cdot \end{array}$$ (5)

For some significance level *α*, *q* is chosen so that across all *R*_*q*_ formed from samples in the subtesting data, 100(1 − *α*)% of these *R*_*q*_ cover their sample’s true origin **s**_0_. This double bootstrapping approach of selecting a threshold *q* to achieve nominal coverage has been studied extensively [32–34]. We call (Eq 5) a 100(1 − *α*)% prediction region, the spatial extension of a prediction interval, constructed to contain the true origin with probability 1 − *α*. As the subtesting data is independent of the subtraining data used to train the model, and these combined training data are independent of the testing data, we ensure the *q* acquired in this manner will produce prediction regions in the testing data with approximately 100(1 − *α*)% coverage.

### Computation

To implement our method in reasonable computing time, we obtained a fine grid 𝓣 by overlaying the U.S. with a 100 × 100 grid of points and retaining only those points that fell over U.S. soil. Treating these points as grid cell centers yielded a finely granular grid 𝓣 of *N* = 6,041 cells, each of dimension 28.0 km north-south by 58.6 km east-west. Performing spatial prediction over 𝓣 required a collective computing time of just under 3 hours using R statistical software and parallelized code distributed across five cores of a 3.6 GHz machine with 60 GB RAM running 64Bit CentOS Linux 5.0. The code is available online at https://github.com/nsgrantham/fungi-identify.

## Results

**Fig 1:**
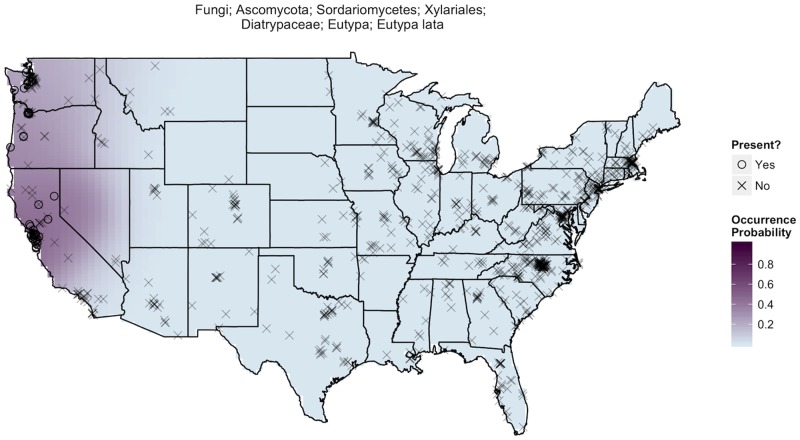
*Eutypa lata* distribution. A map of estimated occurrence probabilities of *Eutypa lata*, just one of our nearly 40,000 fungal taxa, communicates quite a bit about its spatial spread and prevalence. Each° marks the location of a dust sample identifying significant traces of Eutypa lata while an × indicates sample locations where the species was absent. Kernel smoothing produces estimated occurrence probabilities of *Eutypa lata*, where areas with darker purple shading are more likely to produce samples containing traces of *Eutypa lata*. In our 928 samples, *Eutypa lata* was found exclusively in the west near the grapevines on which it depends. This species has a 40–50% chance of appearing in a sample taken in the regions in which is it found.

**Fig 2:**
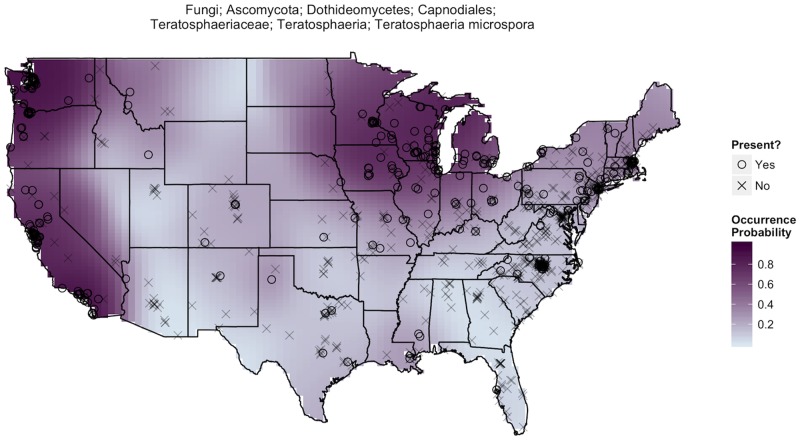
*Teratosphaeria microspora* distribution. The biogeography of *Teratosphaeria microspora* is much different than that of *Eutypa lata* (Fig 1). The darker and more prevalent shading suggests *Teratosphaeria microspora* is a fairly common and widespread fungal taxa. However, it occurs with highest frequency among samples collected from the West Coast and throughout Midwestern regions bordering the Great Lakes.

The sequence-based methods yielded a database of 38,473 fungal taxa with 72.4% of taxa found in < 10 samples, 96.1% found in < 100 samples, and an average of 727 fungal taxa per individual dust sample. Our statistical analyses of these data revealed that many fungal taxa exhibit a high degree of geographic endemism. This endemism is at the root of our ability to make predictions about the geographic origins of samples. For example, consider the geographic distribution of *Eutypa lata* (Fig 1) which was not found in a single sample east of the Sierra Nevada mountain range but had reasonably high occurrence probabilities in samples collected from northern California. A sample with *Eutypa lata*, a common pathogen of grapevines [35], is therefore more likely to have originated from grape-growing regions in the western U.S. By comparison, *Teratosphaeria microspora* is a far more ubiquitous fungus (Fig 2), but it occurs most frequently around the Great Lakes and along the West Coast where there is a > 90% chance its presence is identified in a randomly selected sample. Therefore, the absence of *Teratosphaeria microspora* suggests a sample is less likely to have originated from these regions. Of course, these are just selected examples and ultimately, our ability to statistically assess the origins of samples results from considering the geographic distributions of a much larger portion of the 38,473 identified fungal taxa.

**Fig 3:**
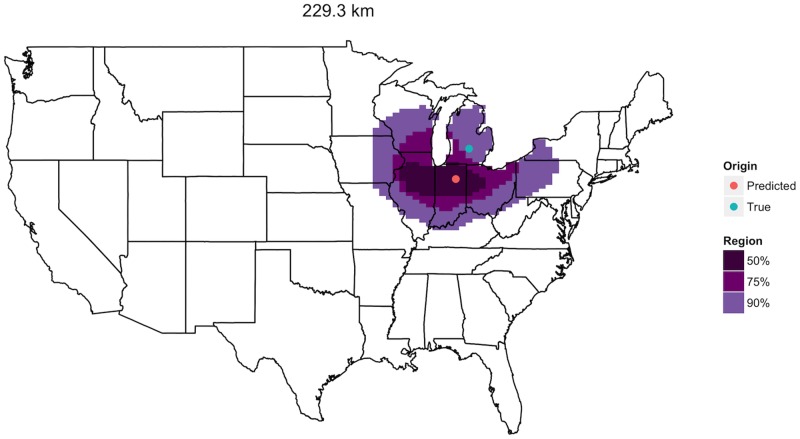
Single spatial prediction. A sample taken in central Michigan is predicted to have originated from northern Indiana, a prediction error of 229.3 km. This distance marks the median prediction error of our method’s 928 total predictions.

Fungal occurrence probability distributions combined with the fungal taxa identified in a dust sample taken in the U.S. allow for prediction of the sample’s most likely geographic origin. Moreover, 50%, 75%, and 90% prediction regions help to capture the shape and spread of the sample’s unique collection of fungal communities by marking regions where the sample is likely to have originated with respective probabilities 0.5, 0.75, and 0.9. Consider a sample taken from a home in central Michigan, USA (Fig 3). Our methods identified a location just 229 km to the southwest in northern Indiana as the sample’s most likely origin. The narrow prediction regions at varying confidence levels show that the fungal community in this sample is characteristic of the region in question, suggesting that samples with similar communities are not expected to originate far from the Great Lakes.

**Fig 4:**
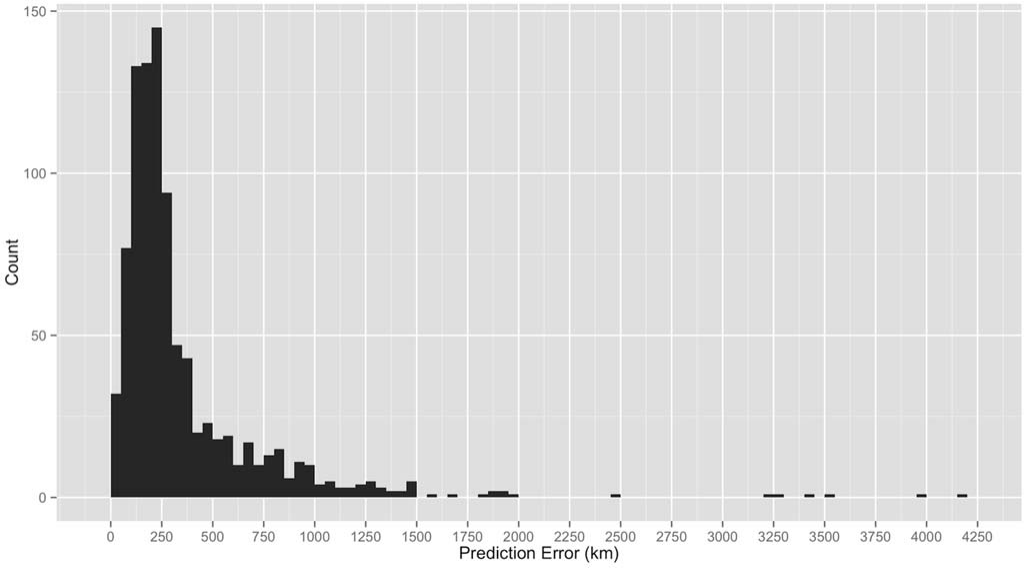
Prediction errors. Histogram of prediction error for *n* = 928 predictions over five-fold cross validation.

Across all 928 samples, median prediction error was 230 km (similar to that in Fig 3) with 5% of samples achieving better than 58 km and 5% achieving worse than 1,039 km (Fig 4, Table 1). The 50%, 75%, and 90% predictions regions formed by the subtesting data retained their respective coverage rates when applied to the testing data. Table 1 summarizes prediction errors and prediction region coverage across several key covariates including the density of samples (sampling intensity), number of different fungal taxa present (fungal richness), daily temperature, daily precipitation, and land type.

**Table 1:** Numerical summary of predictions overall and across several covariates by prediction error (km) and percent of prediction region coverage.

		Prediction Error (km)		Region Coverage		
	*n*	Median	90% Interval	50%	75%	90%
Overall	928	229.5	(57.7, 1038.7)	0.51	0.75	0.89
**Sampling Intensity (within 100 km)**						
Low (≤ 6 locations)	324	278.4	(55.9, 974.9)	0.35	0.62	0.81
Medium (> 6 & ≤ 25 locations)	308	225.9	(54.2, 1039.9)	0.59	0.81	0.91
High (> 25 locations)	296	199.1	(71.0, 1051.4)	0.60	0.83	0.94
**Fungal Richness**						
Low (≤ 543 taxa)	306	343.0	(72.9, 1492.1)	0.51	0.75	0.90
Medium (> 543 & ≤ 814 taxa)	316	223.4	(62.1, 823.9)	0.48	0.74	0.86
High (> 814 taxa)	306	188.6	(54.1, 534.0)	0.55	0.75	0.90
**Mean Daily Temperature**						
≤ median (12.34°C)	464	224.5	(53.9, 1230.7)	0.52	0.74	0.87
> median (12.34°C)	464	235.7	(62.1, 980.4)	0.50	0.76	0.90
**Standard Deviation Daily Temperature**						
≤ median (8.81°C)	464	222.0	(59.4, 977.0)	0.55	0.79	0.91
> median (8.81°C)	464	238.1	(55.0, 1211.1)	0.47	0.71	0.86
**Mean Daily Precipitation**						
≤ median (88.68 mm)	464	226.8	(57.2, 845.4)	0.51	0.74	0.87
> median (88.68 mm)	464	234.6	(62.1, 1244.4)	0.51	0.76	0.91
**Standard Deviation Daily Precipitation**						
≤ median (48.22 mm)	465	248.9	(70.7, 1151.9)	0.45	0.69	0.86
> median (48.22 mm)	463	210.5	(55.8, 979.1)	0.57	0.81	0.92
**Land Cover**						
Developed, Open Space	184	237.5	(67.8, 980.4)	0.45	0.74	0.89
Developed, Low Intensity	277	228.9	(54.6, 845.8)	0.50	0.75	0.90
Developed, Medium Intensity	192	200.0	(54.0, 1745.2)	0.64	0.79	0.88
Developed, High Intensity	59	161.4	(48.6, 1094.3)	0.73	0.85	0.93
Barren Land (Rock/Sand/Clay)	4	371.8	(104.9, 594.5)	0.25	0.25	0.75
Deciduous Forest	52	303.7	(145.7, 1456.4)	0.35	0.60	0.81
Evergreen Forest	32	223.7	(103.3, 922.5)	0.50	0.75	0.91
Mixed Forest	14	221.4	(169.8, 912.6)	0.50	0.86	1.00
Shrub/Scrub	21	222.2	(104.3, 935.1)	0.38	0.76	0.86
Grassland/Herbaceous	16	217.9	(129.0, 1067.8)	0.38	0.88	0.88
Pasture/Hay	48	298.4	(114.2, 878.7)	0.38	0.60	0.88
Cultivated Crops	22	272.5	(73.7, 841.4)	0.50	0.77	0.82
Woody Wetlands	5	663.5	(92.1, 1000.6)	0.40	0.80	0.80
Emergent Herbaceous Wetlands	2	114.9	(93.6, 136.2)	1.00	1.00	1.00

As might be expected, the accuracy of predictions was lower for samples taken at locations without many neighboring locations (low sampling intensity) as evidenced by higher median prediction error (278 km) and poor prediction region coverage (Table 1). Predictions also suffered for samples exhibiting low fungal richness. Differences in temperature and precipitation likely contribute to differences in fungal distributions across the U.S. [22], and we detected slight contributions of these climatic variables to our error distributions. Prediction errors tended to be higher and prediction region coverage lower for locations with relatively stable precipitation throughout the course of a year (below-median precipitation standard deviation) and, to a lesser degree, more variable temperature (above-median temperature standard deviation).

Urban and suburban (i.e., developed) land types had low prediction errors and high prediction region coverage probabilities relative to less developed areas. The accuracy of our predictions also varied depending on the geographic region in question (S1 Fig). Predictions made in the western half of the U.S. (west of -100° longitude) tended to land in close proximity to their sample’s true origin (median prediction error of 190 km). Prediction errors were relatively high for samples taken in the northern states of Idaho, Montana, Wyoming, and the Dakotas, likely due to the dearth of sampling in this region. Conversely, even though the East Coast was very well sampled, predictions were least accurate in a narrow band from New England down to Mississippi.

## Discussion

In short, the accuracy of our prediction model depends on sampling intensity, climatic conditions, and the region being considered. The accuracy of our approach could likely be improved by more thorough sampling in regions of low sampling intensity. The approach may also be improved by deeper sequencing of samples or by sequencing multiple samples from a given source (where such samples exist).

Our results represent an important proof of concept of a novel approach in forensic biology. Based on the dust collected outside homes with a sterile swab we can identify the geographic origin of a sample taken in the United States with a median error of 230 kilometers. More importantly, we can place a measure of certainty on each prediction that a sample originated in a particular place.

**Fig 5:**
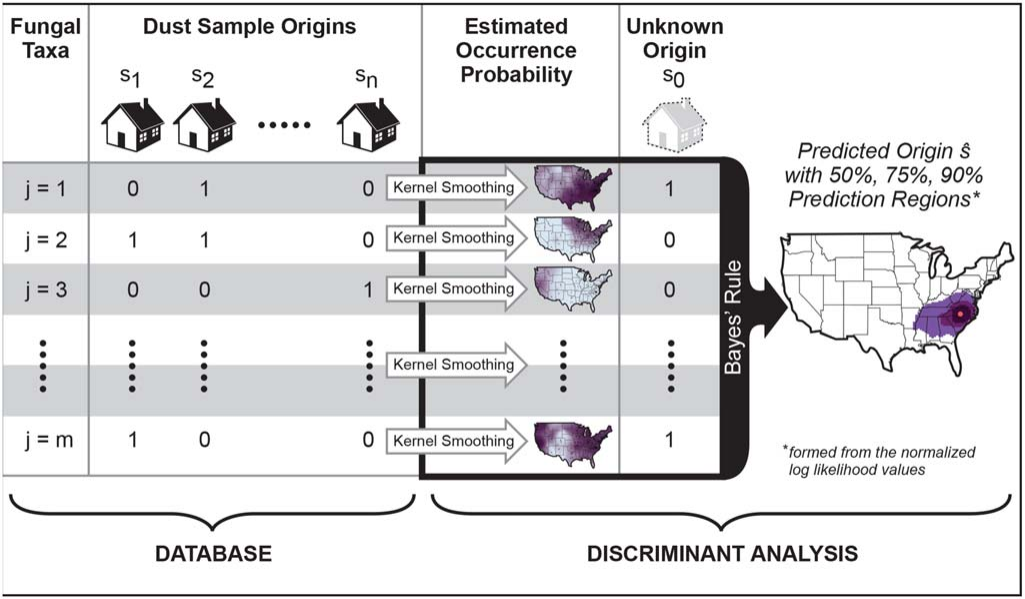
Summary of the spatial source prediction method. Given a database of sequenced dust samples from known origins **s**_1_,…,**s**_*n*_, kernel smoothing produces taxon-specific “hot spot” maps like Figs 1 and 2. Using Bayes’ rule, our method combines these estimated occurrence probabilities with the sequenced taxa data observed in a new dust sample of unknown origin **s**_0_ to identify the sample’s most likely origin $\hat{s}$ (red point). The enveloping prediction regions suggest broader areas where the sample is likely to have originated beyond a single “pin-in-a-map” point estimate.

Moreover, our method is easily amenable to dust analysis by forensic teams. As shown in Fig 5, a new dust sample need only have its fungal communities sequenced before being fed to our spatial source prediction method which compares the new sample against a database of samples from known locations to arrive at a most likely place of origin. Of course, successful applications of this method to new regions will require a priori information on the distributions of fungal taxa across the region of interest—information that can only be obtained by collecting and analyzing reference dust samples to be incorporated into the database.

We were able to predict the origin of samples because many fungal taxa are more likely to be found in some regions of the United States than in others. Many biotic and abiotic factors contribute to the observed biogeographical structure in dust-associated fungal taxa, including differences in host plant communities, climatic conditions, soil edaphic factors, land-use type, and agricultural practices. In addition, biogeographic regions also differ in the composition of their fungi on the basis of historical isolation (and dispersal limited taxa [36]). Better understanding the factors that limit individual fungal taxa may allow us to predict not only the geographic origin of samples, but also other attributes of the area of origin. For example, where the distribution of fungal pathogens of plants coincides with that of host plants, the presence of those fungi can be used as an indication not just of geography but also of farming and land use practices (Fig 1).

The approach described here could be extended in several ways. First comparison of our results to models on the basis of the distribution of plants (pollen), bacteria, or even animal parts will be both possible and informative [17]. Dust has long been known to contain biomass (and hence DNA) from a broad range of taxa, including bacteria and arthropods [1]. Inclusion of data from these other taxa is likely to increase the accuracy of our models. Second, we would also like to be able to identify the origin of many kinds of samples, not just dust samples from houses and we assume that our approach could also be applied to other sample types. The accumulation of dust on other surfaces is an obvious next step, so too is soil. Microscopy-based or geochemical analyses of soil could be useful for identifying the geographic origin of soil samples [37], but these methods are not trivial and their utility for geolocating samples from across the U.S. remains undetermined. Third, to understand the global utility of our approach we will need to consider samples from other regions. The precision of global biogeoprinting will be contingent on the number of species restricted to particular climates and hosts (as in the U.S.), but also to particular biogeographic regions. Finally, here we explore samples from a single region on which biological material has settled. The holy grail of biogeoprinting will be understanding whether not just the origin but also the trajectory of samples can be ascertained. By the same token, it may be possible that useful differences exist among seasons that would allow one to discern not only the geographic origin, but also the timing of dust exposure. In this preliminary study, we could not assess the temporal variability in these fungal communities given that the settled dust accumulated over an indeterminate period of time. However, our data suggest that the temporal variability in fungal community composition is less than the geographic variability. If this were not the case, our approach would likely not perform as well as it has.

## Supporting Information

S1 Fig All 928 predictions produced by the model over five-fold cross-validation. Each line connects a sample’s true (blue) and predicted (red) origin.
(TIFF)
