# Supplementary figures and images for: Fungi Identify the Geographic Origin of Dust Samples
Source: PLoS One. 2015 Apr 13;10(4):e0122605. doi: 10.1371/journal.pone.0122605 (PMC4395444; doi:10.1371/journal.pone.0122605)

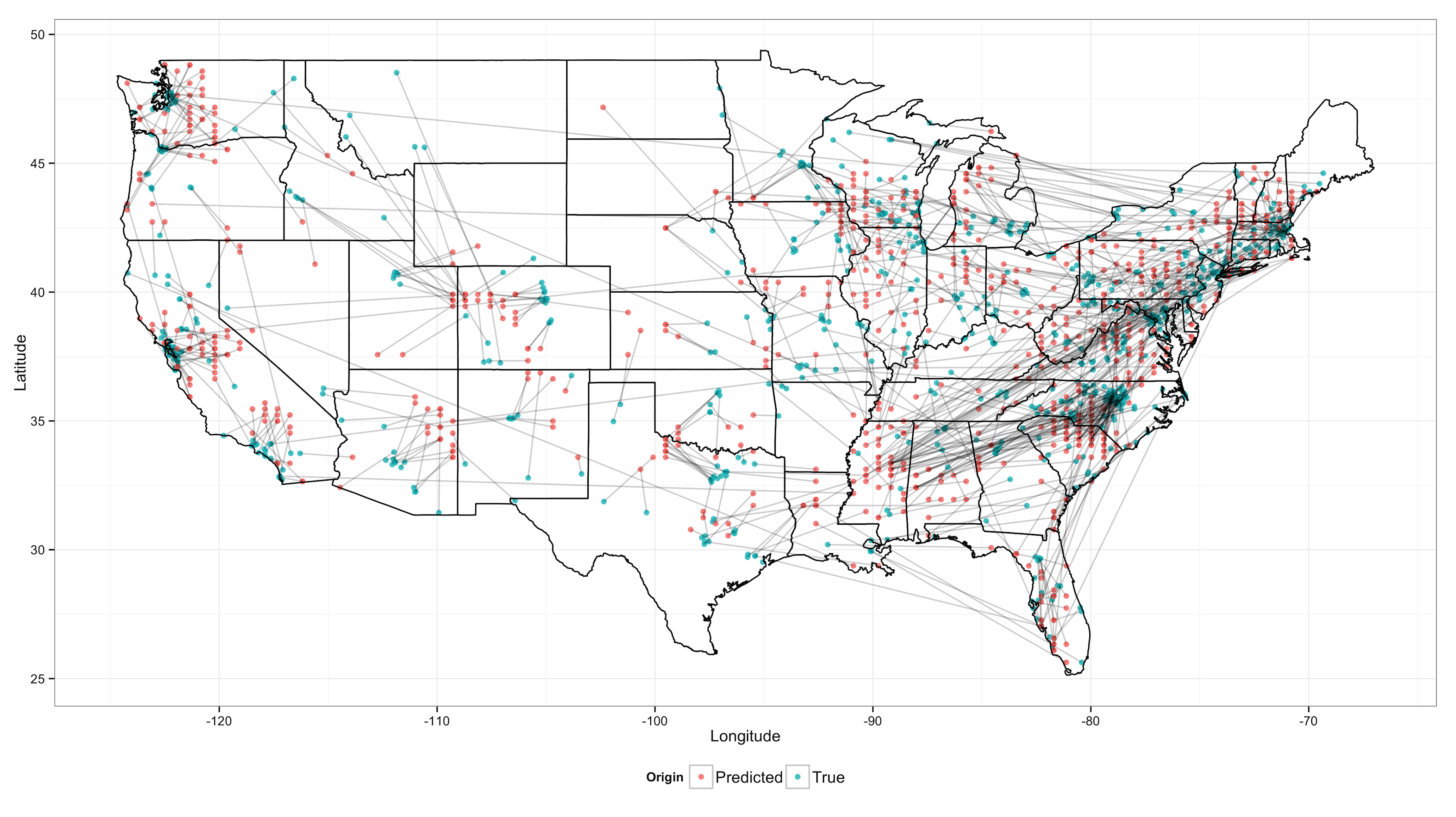

Supplement: S1 Fig — Each line connects a sample’s true (blue) and predicted (red) origin. (TIFF) [file pone.0122605.s001.tiff]
